# The value of cultural diversity: rhetoric and reality: Meeting Report on Fellows Day, International Academy of Intercultural Research 9th Biennial Congress, Bergen, Norway

**DOI:** 10.1186/s40064-016-2456-2

**Published:** 2016-06-27

**Authors:** Milton J. Bennett

**Affiliations:** Intercultural Development Research Institute, Hillsboro, OR USA; Intercultural Development Research Institute, Milan, Italy

**Keywords:** Cultural diversity, Inclusion, Intercultural communication, Cross-cultural relations, Intercultural sensitivity, Ethicality

## Abstract

This report is meant to summarize the discussion themes introduced in the Fellows Day session of the 9th Biennial Congress of the International Academy of Intercultural Research held in Bergen, Norway June 28, 2015. The report also attempts to summarize some of the participant comments made during the session. Because it is a report and not an original research article, descriptions of concepts are intentionally truncated and referencing is restricted to establishing context more than linking to other contemporary treatments of the issue. Further, the summary of participant comments is necessarily selective and may not reflect the complete intention of the commentator. Participant comments were for the most part informed observations or statements of opinion and are therefore not accompanied by formal references. The Fellows Day session was advertised with the following description: Practitioners of intercultural communication and cross-cultural psychology routinely make the claim that cultural diversity is an asset to teams, organizations, and societies. The more research-oriented among us quote studies that have shown correlations of creativity and heterogeneity of teams, profitability and diversity in corporations, and innovativeness and immigration in societies. These studies serve our purposes, but to what extent are they indicative of a general value of diversity? What is the research that fails to show these correlations or that suggests complex mediating factors? How do we integrate our understanding of immigrant assimilation with the preservation of ethnic diversity? How can we extend the idea of preserving diversity to the realm of mergers and acquisitions in corporations? Is the global village generating homogeneity, or is it really, as McLuhan (Understanding media: the extensions of man. McGraw-Hill, New York, 1964) put it, that our neighbors will be profoundly different from us? And is that intrinsically good, or do we need to make it good? These and other questions central to our social future are appropriate for consideration by IAIR Fellows. I propose that we do so in three exploratory phases: *The first exploration is of the rhetoric: what we want to believe, what we sell, and how we support that with anecdote and ideology. I am not using "rhetoric" in a pejorative way here; I mean it more like "narrative" or "value proposition" (which is, by the way, the way the term is used by academic rhetoricians). Thus, the idea is to explore the assumptions we are making in claiming that cultural diversity is a good thing. I hope part of this conversation will position our narrative in the largely post-enlightenment, post-modern Western context that is its home, and that is will also explore how the idea of diversity has or has not emerged in other geo/historical contexts. *The second exploration is a more critical view of our claims, seeking both supportive and contradictory theory and research regarding the value of diversity and/or the value of uniformity. I don't mean to juxtapose "reality" in a positive way to "rhetoric." Rather, I mean to invoke the empirical reality of research as a tool to assess and either support or not our rhetorical claims. I hope we can include both quantitative and qualitative and both descriptive and nomothetic forms of research in our consideration, with the goal of seeing how our claims about the value of diversity stand up to systematic observation. * The third phase is reconciliatory, exploring ways to form a dialectic of diversity and unity that would provide us with a more sophisticated guide to practical action in the areas of education, corporate consulting, and intercultural training. In my practical work as a trainer and organizational development consultant, I have observed that no matter how sterling the value of diversity might be, it needs to be reconciled with the frequently more highly valued need for "unity" — shared goals, common procedures, universal policies, etc. Given our discussion of the day, how might we address both sides of this dialectic in practical, effective, and acceptable ways? In other words, where can we go from here…

## Background

This report is meant to summarize the discussion themes introduced in the Fellows Day session of the 9th Biennial Congress of the International Academy of Intercultural Research held in Bergen, Norway June 28, 2015. The report also attempts to summarize some of the participant comments made during the session. Because it is a report and not an original research article, descriptions of concepts are intentionally truncated and referencing is restricted to establishing context more than linking to other contemporary treatments of the issue. Further, the summary of participant comments is necessarily selective and may not reflect the complete intention of the commentator. Participant comments were for the most part informed observations or statements of opinion and are therefore not accompanied by formal references.

The Fellows Day session was advertised with the following description:

Practitioners of intercultural communication and cross-cultural psychology routinely make the claim that cultural diversity is an asset to teams, organizations and societies. The more research-oriented among us quote studies that have shown correlations of creativity and heterogeneity of teams, profitability and diversity in corporations and innovativeness and immigration in societies. These studies serve our purposes, but to what extent are they indicative of a general value of diversity? What is the research that fails to show these correlations or that suggests complex mediating factors? How do we integrate our understanding of immigrant assimilation with the preservation of ethnic diversity? How can we extend the idea of preserving diversity to the realm of mergers and acquisitions in corporations? Is the global village generating homogeneity, or is it really, as McLuhan (McLuhan 1964) put it, that our neighbors will be profoundly different from us? And is that intrinsically good, or do we need to make it good?

These and other questions central to our social future are appropriate for consideration by IAIR Fellows. I propose that we do so in three exploratory phases:

- The first exploration is of the rhetoric: what we want to believe, what we sell and how we support that with anecdote and ideology. I am not using “rhetoric” in a pejorative way here; I mean it more like “narrative” or “value proposition” (which is, by the way, the way the term is used by academic rhetoricians). Thus, the idea is to explore the assumptions we are making in claiming that cultural diversity is a good thing. I hope part of this conversation will position our narrative in the largely post-enlightenment, post-modern Western context that is its home and that is will also explore how the idea of diversity has or has not emerged in other geo/historical contexts.
- The second exploration is a more critical view of our claims, seeking both supportive and contradictory theory and research regarding the value of diversity and/or the value of uniformity. I do not mean to juxtapose “reality” in a positive way to “rhetoric.” Rather, I mean to invoke the empirical reality of research as a tool to assess and either support or not our rhetorical claims. I hope we can include both quantitative and qualitative and both descriptive and nomothetic forms of research in our consideration, with the goal of seeing how our claims about the value of diversity stand up to systematic observation.
- The third phase is reconciliatory, exploring ways to form a dialectic of diversity and unity that would provide us with a more sophisticated guide to practical action in the areas of education, corporate consulting and intercultural training. In my practical work as a trainer and organizational development consultant, I have observed that no matter how sterling the value of diversity might be, it needs to be reconciled with the frequently more highly valued need for “unity”—shared goals, common procedures, universal policies, etc. Given our discussion of the day, how might we address both sides of this dialectic in practical, effective and acceptable ways? In other words, where can we go from here….

This report includes a summary of introductory remarks and a textual description of comments, criticisms and responses on the topic. The author of this report, Milton Bennett, asserts that he has no conflict of interest related to the topic or the material contained in the report.

## Phase I: The rhetoric of cultural diversity

By way of introducing the topic, this phase of the day will address three questions:

1. What is the paradigmatic basis of *cultural relativity* and what is the implication flowing from that paradigm for the value of cultural diversity?
2. Is attributing value to cultural diversity a particularly Western post-enlightenment, even post-modern phenomenon?
3. Are we experiencing a clash of paradigms, including profound disagreement about the value of cultural diversity driven by different paradigmatic worldviews?

At the end of the Phase I presentation, comments and responses are summarized before moving on to Phase II and III (which were combined in the actual session).

### Presentation

I use the term “paradigm” in the sense defined by Kuhn (1967) as foundational epistemologies that change over time. I like the specific terminology popularized by Briggs and Peat (1984), where they distinguish three major paradigms in physics: Newtonian, Einsteinian and Quantum. I have argued in my recent book (Bennett 2013) that the physics paradigms have crossed over into social science as positivism, relativism and constructivism, respectively. In the following remarks, I will suggest that our understanding of “diversity” is profoundly influenced by the paradigmatic context in which we are doing the understanding and further, that the very idea of “diversity” is dependent on both a post-enlightenment and a post-modern paradigmatic perspective. That the idea of diversity exists in a paradigmatic and cultural context does not make it weaker, but it does demand that we understand those contexts if we wish to argue for its value.

The concept of “science” itself exists in a paradigmatic and cultural context. As a secular, empirical endeavor, science is a result of the empirical investigation in Western Europe that began in the mid-seventeenth century and culminated with the age of enlightenment in the 18th century. Before we examine the idea of diversity in these modern European terms, it behooves us to consider pre-scientific or alter-enlightenment ways of understanding diversity. By “pre-scientific” I do not mean to imply a teleological view of human development, where science would represent an inherently superior position. On the contrary, science simply offers alternative ways of understanding phenomena—ways that certainly are superior in guiding particular activities such as internet communication and space travel, but ways that are not necessarily superior for all understanding. In fact, non-scientific ways of understanding continue to flourish in various human contexts. We need to comprehend these alternative ways of understanding, since they both represent challenges to scientific views of diversity and are themselves examples of diversity.

Pre-scientific paradigms are essentially religious—not in the sense of being religious systems, but in the sense of assuming supernatural causality. In these paradigms, explanations of why things happen are variations on God, gods, or other supernatural forces that cause the events human beings experience. Supernatural forces may operate capriciously, or they may operate according to some set of rules—albeit not empirically verifiable ones. The forces may be susceptible to influence, although special conditions such as the purity of the petitioner may apply to the success of such influence. In any case, the forces that control one’s fate or, indistinguishably, the fate of one’s group, are assumed to be unquestionably real. In contrast, people of other groups are not assumed to be as real. Pre-scientific paradigms are absolutist and they do not support the idea of “perspective.” Consequently, people experiencing the world through such paradigms cannot fathom the idea that other people might have different perspectives—that, in the classic words of Julian Jaynes (1976), they might be hearing the voices of different gods. And if we lack a theory of mind for others, they cannot exist in equal human terms in our worldview; others can only be part of an unfamiliar and thus fearsome place outside human reality.

Of course, non-European forms of intense scientific activity also exist and I have used the term “alter-enlightenment” to refer to their paradigmatic assumptions. Notable examples occurred in the medieval Islamic world and in the Chinese Han Dynasty. I will expand in a moment on the idea of enlightenment science as having successfully reconciled separate secular and sacred domains. But in alter-enlightenment forms of science, the study of natural phenomena remains as a form of sacred worship, meditation, or correct action**—**it does not go down the path of secular reductionism that characterizes European empiricism. For instance, according to Joseph Needham (as reported by Winchester 2008), early Chinese science was hampered by Buddhism and Taoism, both of which disavowed action in the empirical world and perhaps by a Confucian-driven bureaucracy that stressed the forming of human behavior more than the discovery of natural law. In Islamic science, a similar conflation of sacred and secular, of bureaucracy and behavior may have restricted the development of pure empiricism (Cf. Al Ghazalli 2002). In any case, while the alter-enlightenment paradigms do allow a conceptualization of other people, they do not preclude the simultaneous view that one’s own group is “chosen” by God or some other divine force. Thus, by virtue of not being chosen, other people are inherently inferior. While sometimes the others may be valuable, in the end they are expendable.

The implication of this discussion of non-enlightenment based paradigms is that the value of diversity as Europeans commonly think of it simply does not exist in at least some other social and paradigmatic contexts. Those paradigms allow for the definition of otherness as “not us,” but they do not allow the idea that others have perspectives that are different but equally viable to our own. The post-modern concept of “alterity”**—**the construction of identity as a perspective belonging to self in juxtaposition to other perspectives belonging to others**—**does not make sense in these other paradigms. Consequently, while others may be treated honorably and/or hospitably, they nevertheless are not intrinsically valuable because of their otherness. Particular others may have value as trading partners, or as sources of knowledge, but diversity in general is more likely to be seen as simply disruptive. If we are in the business of talking about diversity to people in non-European contexts**—**as many of us are**—**we need to be conscious of our potentially differing paradigms in regards to otherness, lest we (ironically) assume that everyone thinks like us.

The path to our current rhetoric about the value of diversity begins with the Newtonian paradigm and its translation into social science as positivism. After some nasty episodes between the ruling Church and earlier scientists, Galileo and subsequently Newton were successful in arguing that the study of natural phenomena was, in fact, the study of God’s work (Chandrankunnel 2011). They made the crucial case that God did not rule the universe directly (except perhaps in isolated miraculous circumstances), but that the universe was the natural enactment of God’s law. Thus, we could seek to understand the laws (e.g. of gravity) that determine cause and effect without unduly meditating on the will or whims of God(s). In cinematic terms, God became more like the executive producer than the director of the film and scientists could critique the film without incurring the wrath of the distant producer. Because post-enlightenment science was secularized in a way that bypassed the theocratic controls of the time, it was relatively easy to turn the tools of science onto human behavior, as Comte (1966) did in Sociology. By understanding collective human behavior as if it were a natural event**—**that is, objectively**—**it was assumedly possible to discover the laws of such behavior and thus predict and control it. On the individual level, similar assumptions fueled behaviorism and sociobiology.

**Fig. 1:**
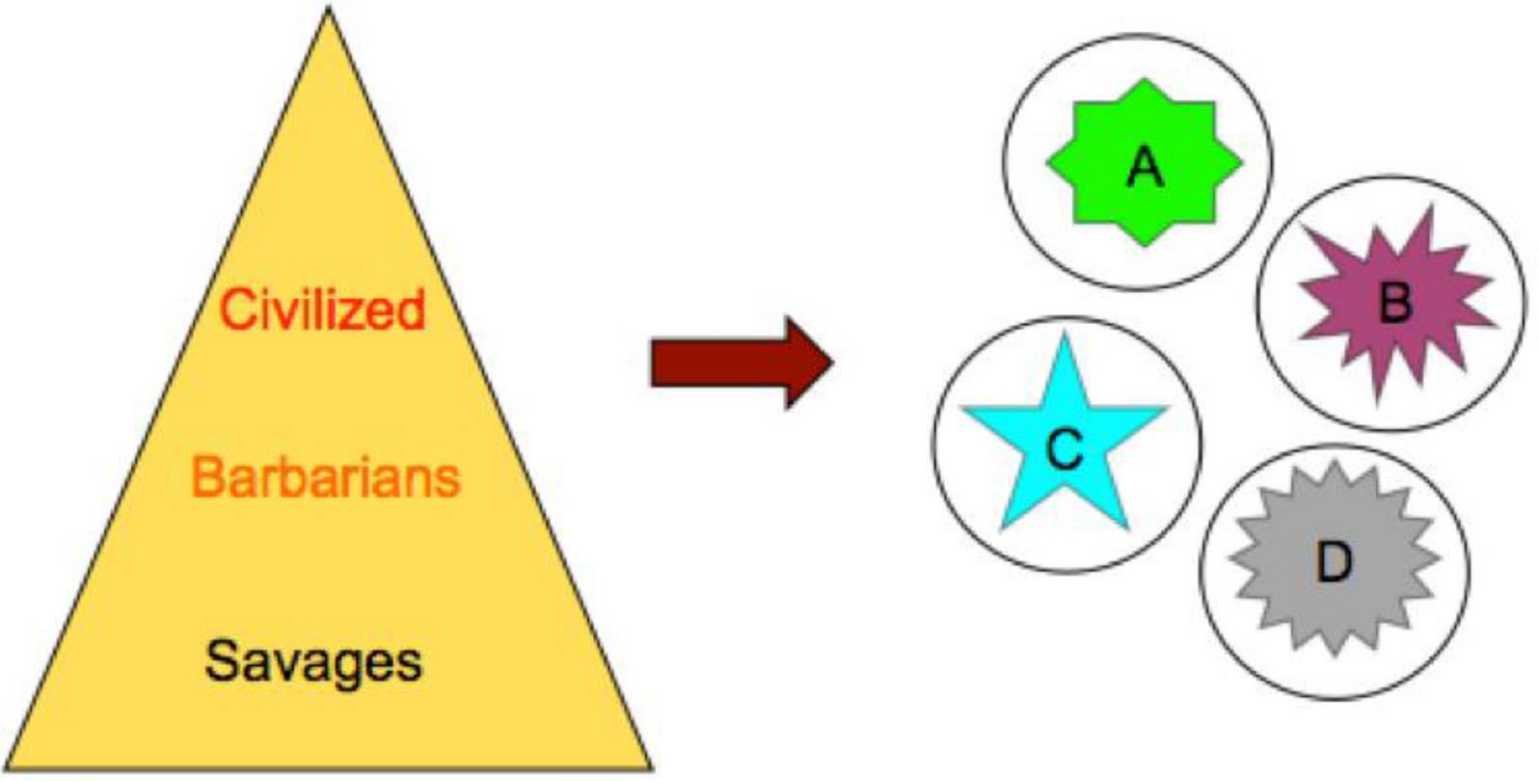
Hierarchy of civilization/cultural relativism

The Newtonian/positivist paradigm was not substantially different than earlier pre-scientific paradigms in its absolutist assumptions; it was just that the absolute reality changed from a more Platonic ideal or transcendent one to a more Aristotelian empirical one. There was still just one correct description of reality, one truth, which in this case is revealed by following the scientific method of investigation. Defined in these terms, a sort of social Darwinism allowed scientifically sophisticated groups to consider themselves as naturally more civilized than other groups who existed at lower levels of a “hierarchy of civilization” (Fig. 1). At the next level down were barbarians who lacked civilization, but who could be brought into civilization through conversion, colonization, or nation building. Below them were savages who lacked complete human characteristics and who could therefore be enslaved or eliminated, if necessary. The Newtonian paradigm did not counteract these earlier historical tendencies to treat outsiders in horrendous ways, but it set the stage for relativism, which did change the game.

The Einsteinian paradigm arguably represents the introduction of perspective into science. Einstein’s theory of special relativity demanded that the relative motion of observers necessarily affected their view of the universe. With this and other assumptions about the relativity of time and space, Einstein’s ideas challenged the prevailing assumption of a static universe that was wholly observable to anyone looking correctly in the same direction. The new Einsteinian universe was more dynamic and, while it still operated according to universal laws, it looked different depending on your observational context. This idea entered social science as “relativism.” The move was particularly evident in anthropology, where it supported Boas’ notion of cultural relativism as an alternative to the hierarchy of civilization (Fig. 1). Cultural relativism holds that every civilization exists in its own context and has its own worldview (thus the notion of “culture” rather than “civilization”). Because they exist in context, cultures cannot be rank-ordered in terms of any universal principle**—**they cannot be more or less civilized. It follows that converting people to one form of civilization (assimilation) is not necessarily the best policy and certainly it is not justified to treat any group as less human.

**Fig. 2:**
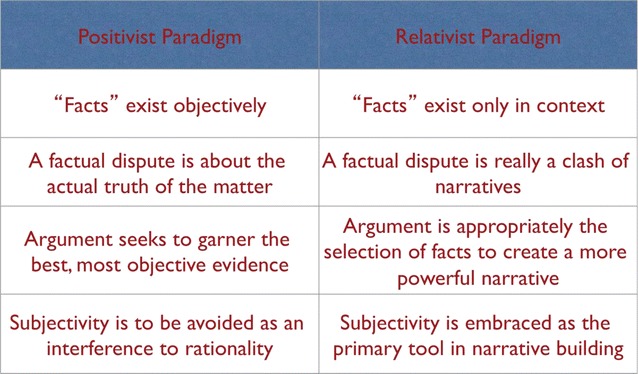
Positivism/relativism

So, the basic idea of diversity is founded on relativism. Cultures, groups, or even individuals are not inherently better or worse than one another**—**they are just different. They may be better adapted to certain environments (e.g. Aleut people are more likely to survive cold weather), but only because the group has developed particular skills, or, in some cases because certain physical characteristics have been selected for (e.g. European milk digestion, Tibetan hemoglobin production). In the area of diversity and human relations in general, the shift from positivism to relativism is fairly complete in European and North American contexts. Among other indicators of the completed shift, there is now a general acceptance of subjectivity in journalism, with “balance” rather than objectivity the goal in reporting; “narrative” is now often stressed over the facts of a matter, so that success in marketing, political positioning, or even career advancement is increasingly “who has the best story (branding)”; positions in policy arguments are attributed to “special interests,” including the special interest of any government agency that might be involved; groups are assumed to be selecting data that support their positions and downplaying data that don’t. These and similar phenomena indicate that *perspective in context* has become an acceptable and even preferable way of explaining social phenomena (Fig. 2).

While none of the aforementioned relativist positions would survive in a more positivist paradigm, their existence doesn’t mean that positivism has vanished. On the contrary, clashes between relativism and positivism or even pre-scientific paradigms are apparently becoming more virulent. Some of these clashes represent the rear-guard action noted by Kuhn (1967). He argues that when paradigms approach the end of their effective lives, they begin to show cracks. People committed to the paradigm try to shore it up by stressing its successes and belittling alternatives. For instance, as the limits of Newtonian-based allopathic medicine approached (e.g. regarding immunology), many health professionals berated non-allopathic methods while redoubling efforts to explain dysfunction through traditional causal relationships. Even though the shift to more holistic, systemic approaches in medicine, particularly in immunology, is now fairly complete, some allopathic physicians continue to denounce any alternative medicine as quackery. Similarly, relativism continues to be attacked by positivists who correctly see it as threatening to more absolutist or universalist explanations of human behavior. In addition, relativism is attacked by supporters of pre-scientific paradigms**—**mostly fundamentalist religious and extremely conservative political believers. Cases of both domestic and international terrorism can be attributed to this paradigm clash (Bennett 2016).

I believe that relativism is now showing cracks, portending its end as a prevailing paradigm in social relations. Following Kuhn’s observation about the likely rear-guard action, supporters of relativism are exaggerating the benefits of the paradigm in ways that are referred to pejoratively as “political correctness.” Typically, in this pejorative sense, political correctness is accompanied by the “PC Police” who foster a climate of fear regarding difference. For instance, I recently consulted with a university that has a website devoted to instructing students how to demand that people use the “correct” set of non-gender specific pronouns with them. (FYI, three such sets were presented, in addition to the traditional “his” and/or “her.”) As the idea of contextualized perspective is extended in many European and North American societies, it is losing its focus on culture and becoming individualized; cultural relativism is giving way to individual relativism. Predictably, individuals are demanding that they can only be understood in their own idiosyncratic terms and that any failure to do so**—**to treat them as members of a group, or to evaluate them in terms of a normative standard**—**constitutes disrespect. The move to individual relativism obviates the very idea of culture and thus the notion that cultural variation can be valuable. Ironically, individual perspective is being rigidified into an absolute position.

As the relativist paradigm deteriorates, the value of diversity is unlikely to be realized through its assumptions. As originally construed in relativism, the value of cultural diversity would derive from variations in how groups adapt to their environments. For instance, if my group is adapted to fast problem-solving and your group is adapted to reflective analysis and assuming that as individuals we can enact this group pattern, then our working together on a project has obvious added value. However, the advantages of diversity do not occur automatically and diversity in and of itself can be a severely disruptive force in organizations and societies. One way this disruption can occur is when relativism is taken to the extreme of political correctness, as mentioned above. In a climate of fear regarding difference, people are reluctant to discuss different culture-based skills for fear of stereotyping either themselves or others. Since cultural differences do not go away just because they are not discussed (cf. Hofstede 1984), they become impediments to effective coordination. In such a politically charged environment, differences may also become associated with “identity politics,” fueling unhealthy competition for resources in both organizations and larger societies.

The impending paradigm is based on the now prevailing philosophy of Quantum mechanics in physics, which takes the form of “constructivism” in social science. While not yet well-established in social science disciplines (many of which are still trying to establish credibility in positivist terms or struggling to meet the excessive demands of relativism), constructivism offers some clear advantages in construing the value of cultural diversity. Added to the relativist notion that facts exist in context, constructivism demands that the observer of data take responsibility for the construction of the context that discriminates the data in the first place. For instance, a *relativist* view of IQ is that the indicators of intelligence are likely to vary by cultural (and other) context and so one can not use IQ as a universal standard. The *constructivist* view of IQ is that it is the result of our inferring a cause for certain consistencies we have attributed importance to in how people respond to various measures we have constructed (Gould 2012). The assumption that we are responsible for constructing a version of reality does not thereby make the construction less valuable. For instance, IQ could continue to be a useful tool in predicting performance in some situations. But we would be more attentive to which situations were appropriate to its use**—**not just in the relativist sense of “unbiased,” but in the constructivist sense of “consistent with our purposes.” To continue with Gould’s argument, if the original purpose of defining “intelligence” was to guide educational intervention, does rank-ordering people in terms of IQ continue to serve that purpose? It is constructivist to take responsibility for asking this question and for creating the context in which an answer is given.

**Fig. 3:**
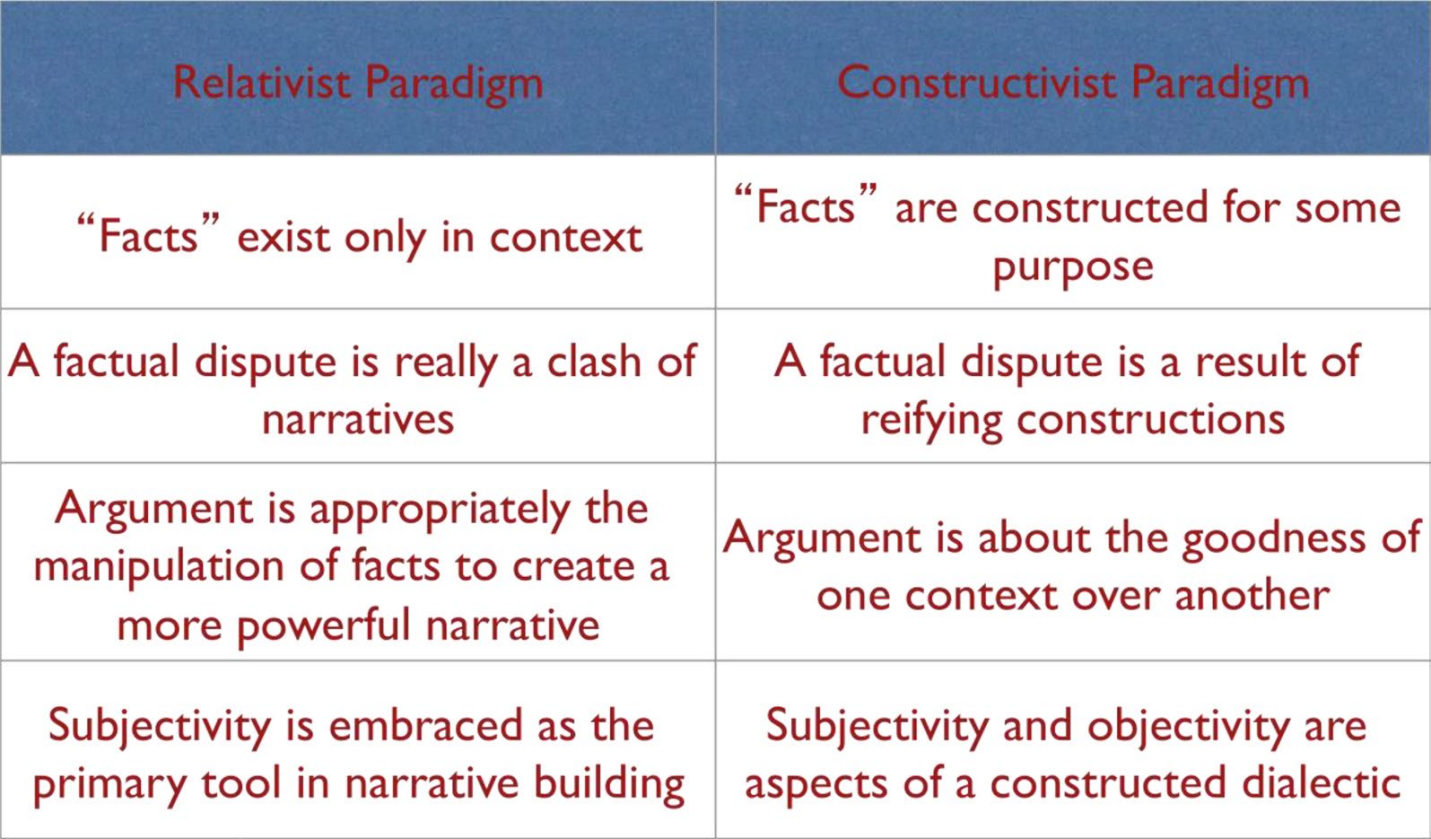
Relativism/constructivism

Other indicators of the shift from a more relativist to a more constructivist paradigm are (1) the shift from assuming that facts exist in context to recognizing our role in creating those contexts; (2) the recognition that differing narratives are not different versions of truth, but more like different versions of reality; (3) and that therefore argument is neither about the truth of a matter or about whose version of truth will prevail, but it is about what version of reality we wish to live in (Fig. 3). The implication of this paradigm shift for valuing diversity is that our focus is shifting from listing cultural differences (as if those differences were intrinsically valuable) to determining ways that differing experiences of reality can be coordinated towards some agreed upon goal.

**Fig. 4:**
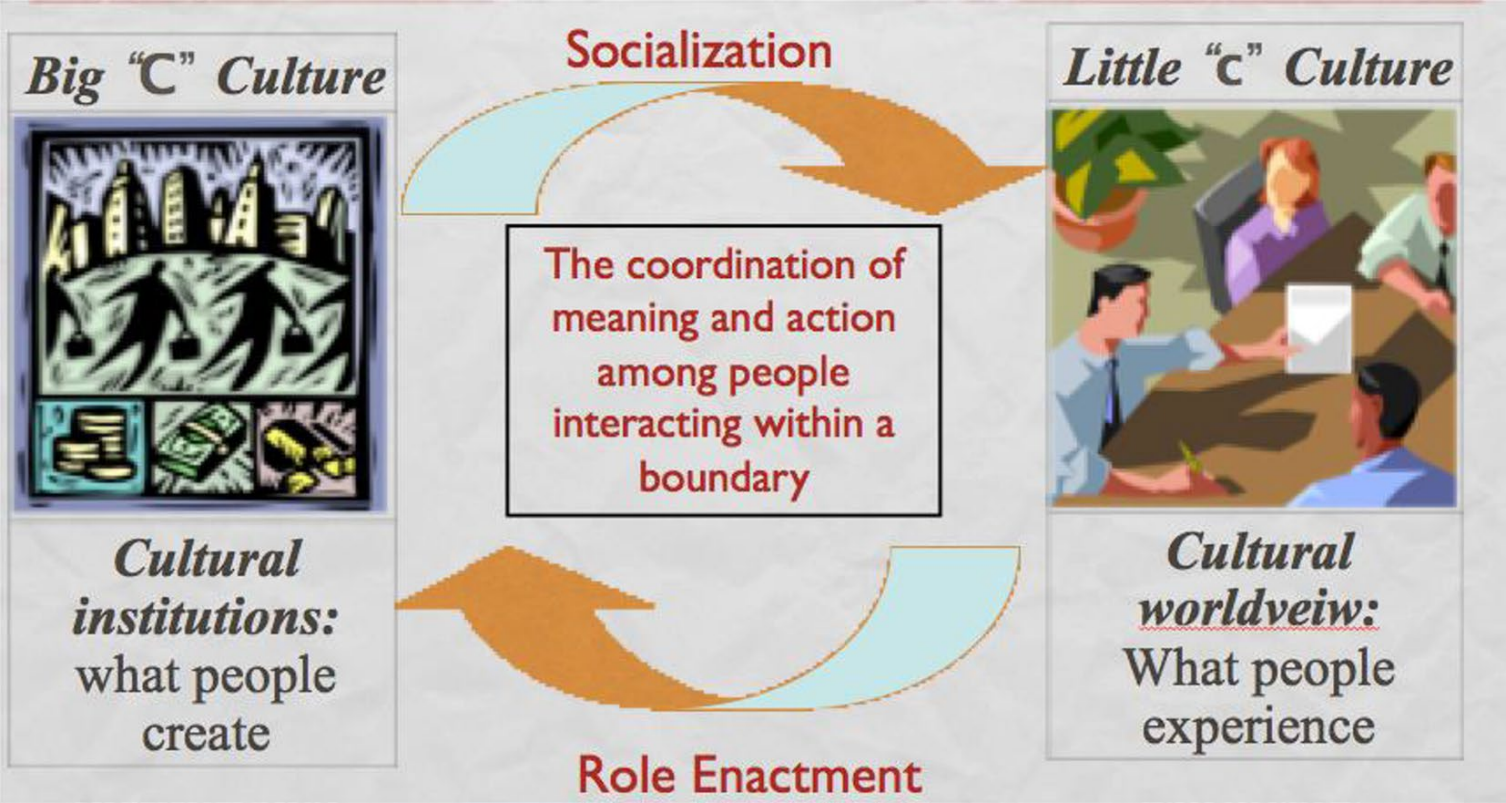
Dialectic of culture

To think in these more processual terms, we need to begin with a non-reified definition of culture such as “the coordination of meaning and action among a group of people defined by a boundary” (Fig. 4). This constructivist definition is similar to some of the ideas of “culture” used by E.T. Hall and Gregory Bateson in their original definition of intercultural communication. In this definition, “culture” is not a thing, but rather an action we undertake. The action of coordination implies purposefulness**—**moving towards some more or less agreed upon goal. The coordination occurs within a constructed boundary, so that, for instance, Europeans could be coordinating themselves within the European Union boundary towards the goal of maintaining peaceful international relations, while they are simultaneously coordinating themselves within national boundaries for the purpose of maintaining economic well-being and distinct national identity, while they are simultaneously coordinating themselves in provincial units for the purpose of maintaining social services, while they are simultaneously coordinating themselves in professional groups for the purpose of maintaining professional standards, etc. Importantly, these boundary conditions need to co-exist as constructs. If we shift to thinking of them as systemic terms**—**super-systems and sub-systems**—**we are shifting back into a relativist paradigm where contexts have an a priori existence. In the more constructivist view, systems only exist insofar as we maintain the definitional boundary**—**a kind of social application of the Quantum principle of “observer dependence.”

**Fig. 5:**
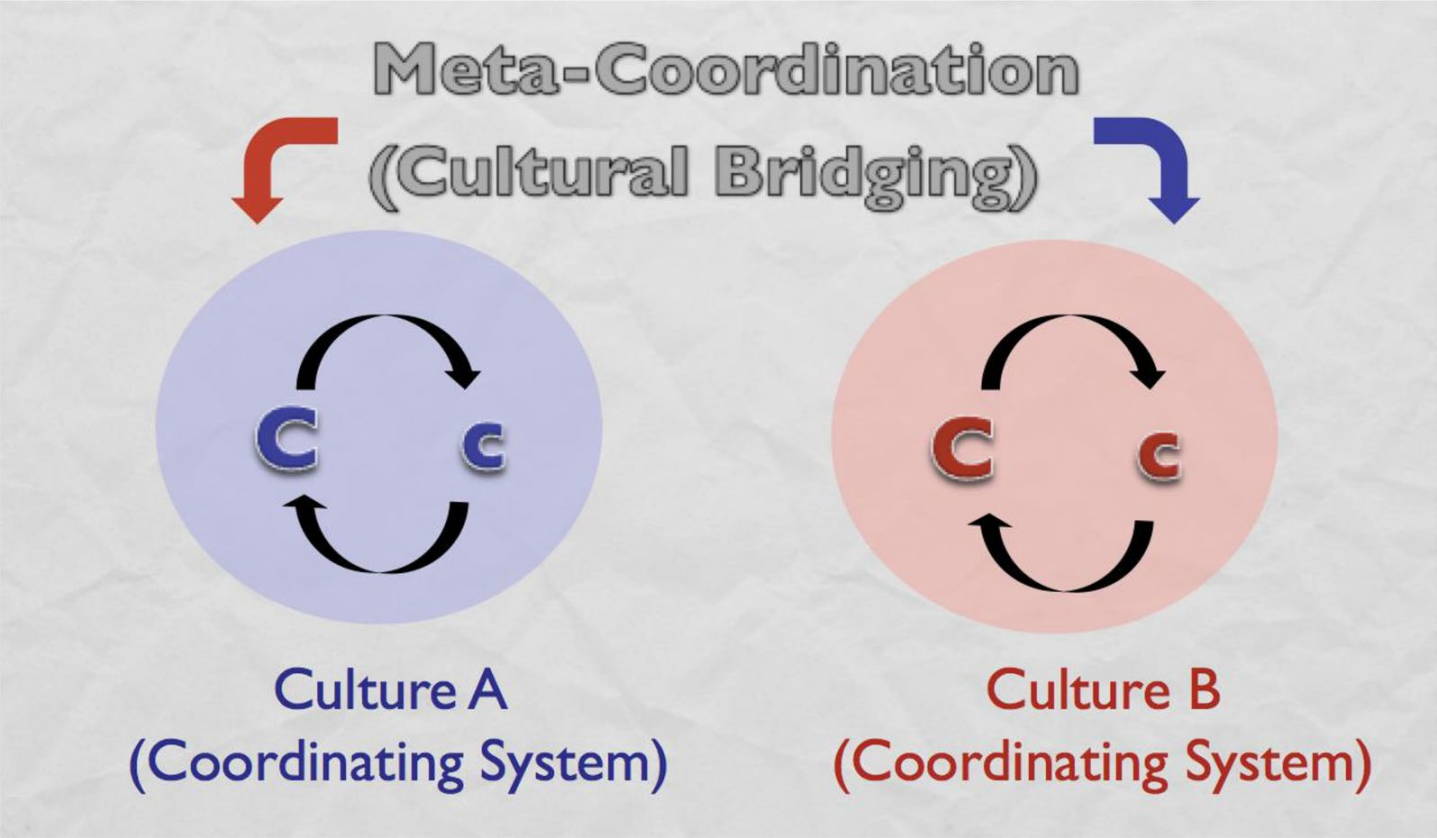
Intercultural meta-level

Following this definition of culture, a constructivist definition of intercultural communication is “the coordination of meaning and action across coordinating systems”—a “meta-coordination” (Fig. 5). Why would we want to do that? On a tactical level, meta-coordination is necessary on multicultural teams and in multicultural organizations or societies to get people moving in more or less the same direction *without eliminating the cultural differences*. In other words, it is the alternative to assimilation. Assuming that assimilation is not that effective even when it’s sincerely attempted, this is a way of acknowledging the ongoing influence of differing socialization in a way that is both respectful and also effective. But more importantly, meta-coordination (what I call “adaptation” in the *Developmental Model of Intercultural Sensitivity*) is the key to deriving value from diversity. Obviously, if we seek to eliminate cultural diversity through assimilation, we are not supporting the potential value of that diversity. So we need to maintain cultural differences, both in a way in which the differences can be coordinated towards some end and also in a way in which the differences can synergize to generate value. For instance, returning to the example of culture-based preferences for problem-solving or reflection and assuming (as we have been) that the differences (1) will not go away in an assimilative context and (2) will not automatically be used towards a mutually desirable goal, the need is to intentionally coordinate the differences towards a goal. In most cases, this assumes that the people involved have a modicum of cultural self-awareness and that they can either themselves shift to a meta level to coordinate their differences or that a skilled facilitator (mediator, coach, trainer, group leader, etc.) can supply the meta-level in interaction with them.

### Discussion (A textual compilation of Phase I participant comments)

#### Criticism

The idea of “culture” itself may be a predominantly Western notion. This would indeed be the case if culture was a construct enabled by the relativist paradigm and if relativism was a particularly Western notion. In support of that view, the pre-scientific or alter-enlightenment paradigms that pervade non-Western societies do not necessarily behave as described by Kuhn, either in the sense of scientific revolution nor in the sequence suggested in the introduction. Those paradigms are for the most part absolutist (truth-based) and/or universalist (all encompassing). So, while the notion of “context” might exist (as it does, apparently, in Japanese and probably other Asian cultures), the idea is defined in perspectival rather than assumptive terms. Kurasawa’s *Rashoman* may be representative of perspectival view of context. Shifting perspective does not necessarily yield an appreciation of the assumptive worldview differences assumed in cultural relativism. And indeed, Japanese and other Asians with an assumedly more developed sense of context do not seem to be systematically less ethnocentric. So there is a good case to be made that “culture” as we currently think about it in Western contexts is uniquely due to the shift from Newtonian to Einsteinian paradigms and its manifestation in social science as relativism.

#### Response

The possible fact that culture is a predominantly Western notion does not make it useless, even in application to non-Western contexts. Apparently our history is filled with examples of diffusion and appropriation of ideas from one culture to another. Why not the idea of “culture” itself? Maybe it’s as useful a concept as the Buddhist system of mindfulness, the Chinese theory of energy meridians, or Mesopotamian idea of writing. In a constructivist paradigm rendering, constructs can be evaluated for their usefulness to a purpose rather than their nativity to a particular social context. We should resist being forced by the PC police into an extreme relativism position where no idea can be applied across cultural contexts, including, as ironically they do, the idea of “culture.”

#### Criticism

The idea of “culture” may be the product of the false dichotomy of individualism and collectivism. Once that duality is established, there needs to be something that allows individuals to act in groups and we (Westerners) created “culture” for that purpose. So the reason culture has not originated as a concept amongst some other social groups is not that they lack the paradigm of relativism, but rather that they lack the dichotomy of individualism and collectivism. Further, we dichotomize groups in terms of their being more individualistic or collectivist, when in fact we all are both.

#### Response

The observation that individualism/collectivism is a constructed idea is assumed in a constructivist paradigm, whether it is treated as a dichotomy or as a continuum. And in that paradigm, the important question is, “Is it useful to make the distinction?” Certainly Triandis and others have shown that it *is* a useful concept in our understanding of how people organize their experience in the world differently. So perhaps the criticism should be about reifying the concept; that is, treating people as if they really were individualistic or collectivist. Reification leads us down the path of classifying people in ways that simplify (stereotype) them and in terms that the people themselves may reject. Instead, we can take responsibility for our observational distinction and use it to understand differences in the coordination of meaning and action, not to label people or cultures as this or that.

#### Criticism

The idea of “culture” may neglect *power*. Critical theory generally suggests that human interaction is a power play of domination and oppression. People who dominate (the dominant group) make the rules and impose them on others, who are typically oppressed by them. The observational categories of racism, sexism and other “isms” previously or yet to be defined refer to this process, both in individual and institutional terms. This critical focus on power is a robust line of theory and research in intercultural relations (notably Martin and Nakayama 2012).

#### Response

The observation of this power dynamic is *not* a silly exercise of political correctness; in fact, people really do thrive on privilege and they really do suffer and die prematurely from oppression. One could even argue that cultures in general are in an existential competition with one another for dominance in defining reality. But that doesn’t mean that people can not also be observed in relatively power-neutral worldview terms; a separation of ethnographic from normative. In a constructivist paradigm, the observations we make are chosen through the contexts we create. When we create the context of relative power, it allows us to observe interactions in terms of dominance and oppression. When we create the context of worldview differences, it allows us to observe interactions in terms of collectivism/individualism and other constructed etic categories. Is one way of observing correct and the other not? Put this way, we probably would agree that both ways of observation are useful and even crucial to our mutual survival. If we don’t understand power, we fail to appreciate the experience of privilege and disadvantage. And if we don’t understand culture, we fail to appreciate alternative ways of being human. One could even argue that the each way of observing is necessary for the other (Cf. Mendoza 2005; Alexander et al. 2014).

## Phase II and III: Deriving the Value of Cultural Diversity

The second and third phases ended up combined. There was not much new research reported specifically on the value of diversity and the discussion went more directly to implementation and the obstacles to implementation of valuing diversity efforts. The questions addressed were:

1. What are some examples of attempts to affirm the value of cultural diversity with empirical evidence?
2. How might an intercultural/cross-cultural approach to diversity address some of the issues of racism currently evident in the US and EU?
3. What are other ways that we might make our work more efficacious in the current global environment?

After the Phase II/III presentation, a final textual summary of comments is included.

### Presentation

A compilation of research on work teams reported by Nancy Adler (2001) shows that multicultural teams are either more or less effective in completing a creative task, compared to monocultural teams. This is an important finding, since people do complain that cultural diversity does *not* add value**—**it’s just a lot of trouble. According to the team research, they’re right, at least part of the time. When they are wrong, when diversity clearly adds value to team performance, the determining factor is leadership. If the leader of a multicultural group acknowledges cultural difference and takes the time to establish it as asset, the team outperforms the monocultural team. But if the leader suppresses cultural difference, usually in the name of the overriding corporate culture, the differences don’t go away**—**they just become impediments to performance.

In general, leadership appears to be the key to deriving the value of diversity. The Harvard Business School professor Rosabeth Moss Kantor (Moss Kanter 1995) has argued that, in the 21st century, all global organizations will have *access* to cultural diversity; but the companies that will thrive in that environment are those who can turn access into an *asset*. And this, she suggests, is largely a leadership issue. That idea is supported by the ongoing HBC Leadership Initiative, where over 1300 entrepreneurial leaders showed a common pattern of being particularly attentive to their own context and able to shift into different contexts (Mayo and Nitin 2005). In other words, the leaders assessed in the HBC research appear to be able to create a meta-level on cultural context**—**a core intercultural communication skill. And it has certainly been my experience as an organizational consultant over several decades that successful diversity efforts must start and be maintained by strong leadership.

The impediments to implementing efforts to derive value from diversity are legion. The main impediment and the one that probably receives the least attention, is the way that we define “culture” and “cultural diversity” in the first place. Positivist and other absolutist approaches to these definitions inevitably yield ethnocentrism, because the very idea of culture cannot exist in those paradigms. Cultural diversity may be treated as non-existent or irrelevant (*Denial* in the *Developmental Model of Intercultural Sensitivity*), or differences may be treated as existential threats**—***Defense* in the DMIS.

**Fig. 6:**
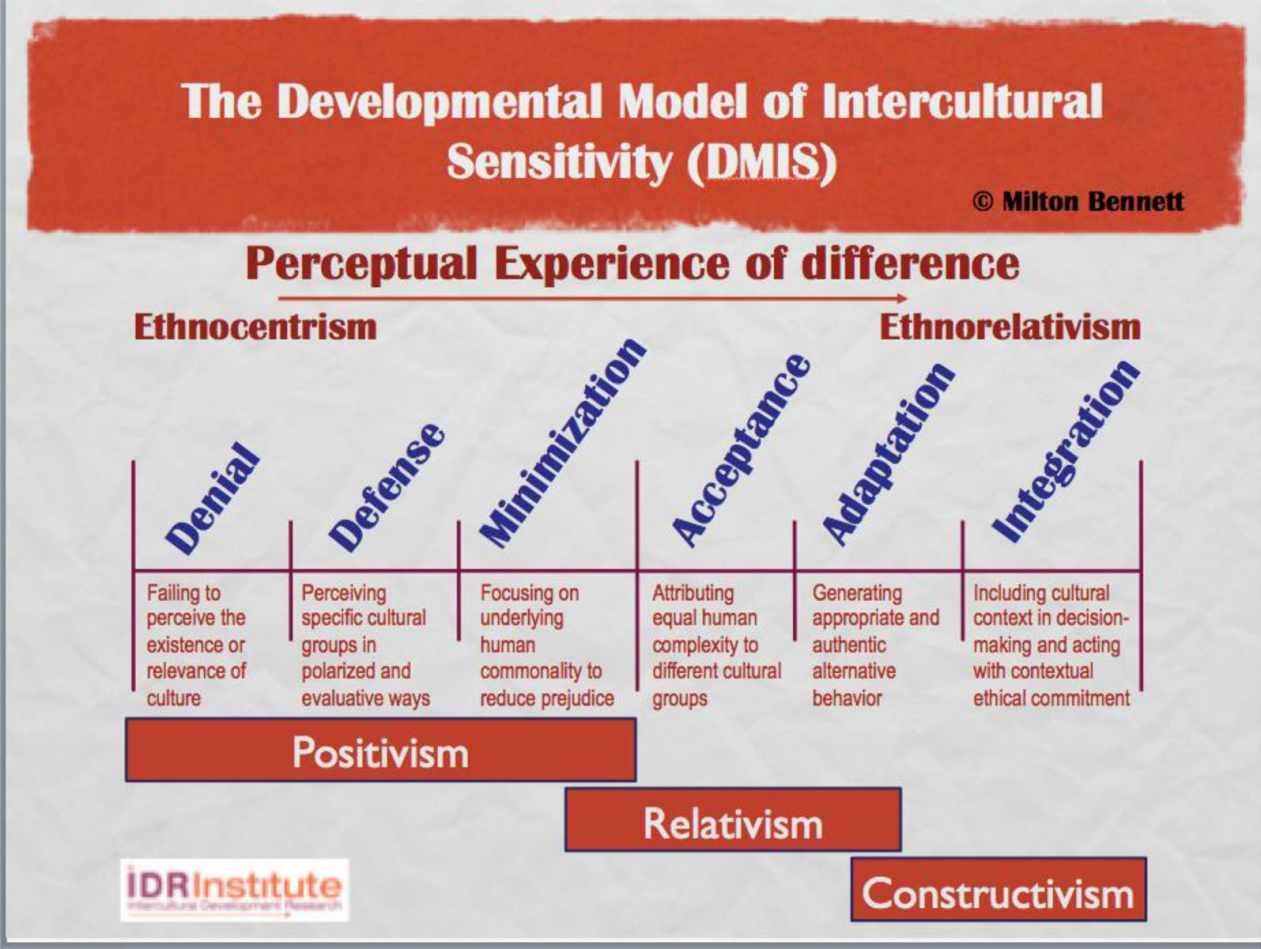
Paradigms of intercultural sensitivity

In the subtlest form of ethnocentrism**—***Minimization***—**cultural differences are seen as surface variations on universal human attributes or values (Fig. 6). The discovery of common humanity is often taken as the “cure” for racism, sexism and other manifestations of Defense. But, ironically, it is just shifts the form of ethnocentrism from overt polarized judgment to covert assumptions about universality of one’s own experience in the world. In terms commonly used in diversity work, Minimization changes racism, sexism and other isms into privilege. Even the *Acceptance* of cultural difference may not support effective programs, since Acceptance can be associated with the extreme relativism of political correctness and thus create more of a climate of fear than a climate of respect for cultural diversity. In DMIS terms, the developmental position most likely to support valuing diversity efforts is *Adaptation*. That position is located in a more constructivist paradigm, where the viability of alternative ways of being are acknowledged and consciousness is available to create meta-levels on cultural contexts. Even then, the efforts may fade away unless they finally emerge from *Integration*, which is the position where previously conscious efforts at valuing diversity become unconscious**—**that is, they become habitual for individuals and institutionalized in organizations.

Another major impediment to implementing diversity work is the issue of *ethicality.* In any of the pre-scientific paradigms, ethicality is straightforward. God(s) or their messengers tell you what is right and you either do that or you don’t and suffer the consequences. Sometimes the alter-enlightenment paradigms (e.g. Islamic science) work similarly, or sometimes they incorporate a more complex set of guiding principles (e.g. Chinese Buddhism). In post-enlightenment societies, ethicality can also be a straightforward matter: a positivist paradigm encourages absolute ethical positions based on authority, although not necessarily a supernatural authority. Obviously, these ethical positions are consistent with ethnocentrism**—**I know what is right and it’s right for you, too (since we all live the same absolute reality).

Ethical ambiguity begins with the acceptance of cultural difference. If we really believe that people can experience the world in different ways that are “not bad or good, just different,” and if part of people’s unique experience is in terms of a particular set of values, then it would be consistent to accept all viable value systems as being equally good. But we do not and we should not. Unfortunately, the alternative generally is to return to ethnocentrism (at least the Minimization form) and claim that despite superficial differences in cultural beliefs and behavior, there really are underlying universal values that apply to all people. It doesn’t matter if the underlying values are “sensitive” ones such as human rights, gender equality, empathy, or “realistic” ones such as social privilege, male dominance and self-interest. It is the assumption of universality that makes them ethnocentric.

In paradigmatic terms, the problem is relativism. Cultural relativisim generates the idea of cultural context, but it does so with the continuing Einsteinian assumption that a universal underlying reality exists despite our sincerely different perceptions and experiences of it. As long as there is no contact or conflict among cultural contexts, the superficial assumption of relativity can hold. But in the case of conflict and the necessity of taking some action for or against a position, superficial cultural relativism generates paralysis. Any action is disrespectful of cultural difference, one way or the other. And since no action is itself an action in conflictual situations, things may devolve into allegations of insensitivity on all sides. The alternative to paralysis, without changing paradigms, is to invoke the assumed underlying reality and thus, to return to ethnocentrism.

**Fig. 7:**
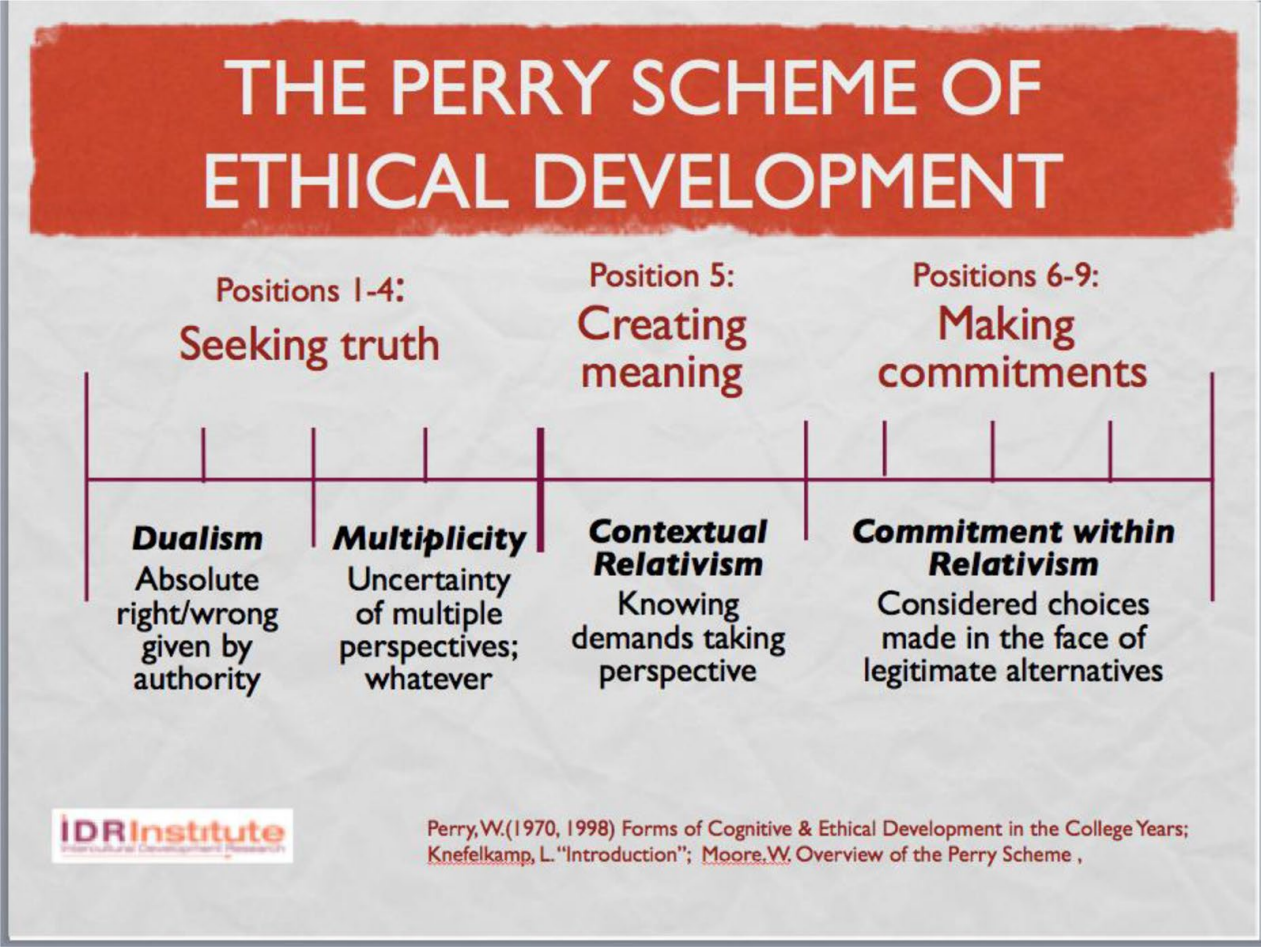
The Perry ethical scheme

Changing paradigms opens up other possibilities of dealing with ethical conflict respectfully. One such constructivist model of ethicality was posited by William Perry (1970) and has been modernized by Lee Knefelkamp (1998). Their model addresses exactly the ethical problem of relativism (Fig. 7). In the developmental scheme, Dualism is the default condition of absolute right and wrong given by some authority. That position may give way to Multiplicity, where the truth is occluded by bias and limited perspective. But both of those positions are “seeking truth” in the sense that there is an underlying (or transcendent) reality that could guide ethical decisions. In paradigmatic terms, the movement from Dualism to Multiplicity is from positivism to relativism. And similarly, in both paradigms the demand for action is a matter of seeking truth.

The alternative to this default method of ethical judgment is contextual relativism. That developmental position demands that we take the perspective of others; it calls for empathically participating in others’ reality, including taking their ethical position on the conflictual issue. In paradigmatic terms, contextual relativism is based in constructivism, in that it assumes that alternative viable realities exist and that we can empathically have access to them. Then, only after having experienced our opponents’ world, can we formulate an ethnorelative action**—**Commitment in relativism. Such an action may look the same as one taken from an ethnocentric position, but it is not; it is respectful of the full humanity of others, including their differing viable values. The world *could* be the other way, but we can choose that it should not be. So, even if we were to decide to violently stop another person or group from doing something, assuming that we had empathically engaged them first, we would be doing so from a considered choice rather than an immutable conviction. This exercise of ethical consciousness is enabled by a constructivist epistemology and necessary for maintaining a climate of respect for diversity.

### Discussion (A textual compilation of Phase II/III participant comments)

#### Criticism

We are living in a perilous time of culture clashes and may be moving backwards in terms of our appreciation of cultural diversity. Massive migration and asylum-seeking in Europe, the Middle East and Africa are overwhelming social services and fueling fascist reaction. Hate crimes are on the increase, both as manifestations of personal bigotry and as forms of domestic terrorism. Inequity is increasing and is exalted by those who are benefiting from it. Women face not only the glass ceiling, but the “glass cliff.” Worse, violence against women continues to exist and is openly justified in political and religious terms by many. Successful efforts at building multicultural societies, e.g. Canada and Germany, are under fire for their very success**—**creating a relatively safe environment for diversity. More than just a clash of cultural norms, we are facing an existential competition for the very definition of social reality. (Author’s note: a reviewer of this report disputes the accuracy of this statement about Canada and Germany.)

#### Response

There are several things we should be doing to counteract the conditions of existential culture clash. One is to implement the well-known Allport/Pettigrew condition for beneficial cross-cultural contact: equal power. As long as inequity exists at the level it does now, exacerbated by economic greed, sexism and xenophobia, cross-cultural contact will yield increased stereotyping and reduced tolerance. At least we should emphasize programs such as student and worker exchange that tend to meet the conditions and actually decrease prejudice.

In terms of the “clash of civilizations” sometimes attributed to current relations between some Islamic groups and others, we should remember that the Crusades are over. One of the most important aspects of the enlightenment in Europe was the reconciliation of sacred and secular that allowed science to flourish without destroying religious belief. While other cultures and religions will not and should not duplicate European history in this regard, we all need to consider how religious and secular experience can co-exist in our societies. The history of theocracies (like that of dictatorships, or absolute monarchies) does not speak well of their fitness to an equitable global environment. It is unlikely that the “end of history” is democracy, or market-driven capitalist systems, managed economies, or any other governing system currently practiced. But it is clear that whatever system we create is one that must accommodate people who are experiencing reality differently and one that can derive value from that fact.

#### Criticism

It may be that the “melting pot” is not as dead as we thought. At least the idea of assimilation seems to be lingering in societies as a vaguely defined goal for acculturating migrant and refugee populations.

#### Response

To counter this discredited ideal, we need to be making a better case for bilingualism and biculturality. The neurological evidence is building for the benefits of these conditions and we need to make the argument that the extension of those benefits can accrue to multicultural societies. The deeper issue is how to reconcile unity and diversity. Too often, these positions are juxtaposed, when if fact every organization and society needs both unity to generate focus and diversity to generate innovation. Our work in intercultural and cross-cultural training or education needs to stress this kind of deep development, rather than dwelling on superficial cultural differences that do not really make a difference to whether we can live together in diversity.

## Conclusion

In sum, the presentation and discussion of the Fellows day suggested at least the following conditions in approaching the value of cultural diversity.

1. A relativist understanding of cultural context that is clearly differentiated from positivism.
2. An avoidance of the political correctness extremes of relativism, while preserving the idea that cultural groupings and identities are inherently respectable.
3. A reconciliation of critical and descriptive worldview approaches to diversity, so that we can talk about power/oppression and worldview differences in a separate but complementary way.
4. An understanding of the role of leadership in establishing a climate of respect for cultural diversity.
5. A constructivist understanding of “cultural experience” and “intercultural relations” that allows us to empathize deeply with others while preserving our own identities and ethical commitments.
6. A recognition of the developmental aspect of intercultural sensitivity and ethicality, so that we can approach living together in multicultural societies and the gobal village as an ongoing adaptation to the social environment we are creating.
